# Genome-wide analysis of promoter and enhancer usage in hematopoietic stem cell differentiation

**DOI:** 10.1186/1756-8935-6-S1-P53

**Published:** 2013-03-18

**Authors:** Annarita Miccio, Clelia Peano, Oriana Romano, Guidantonio Tagliazucchi, Luca Petiti, Ingrid Cifola, Ermanno Rizzi, Marco Severgnini, Silvio Bicciato, Gianluca De Bellis, Fulvio Mavilio

**Affiliations:** 1 Dept. of Life Sciences and Center for Genome Research, University of Modena and Reggio Emilia, Modena, 41125, Italy; 2 Institute of Biomedical Technologies, National Research Council, Milan, 20132, ltaly; 3 Department of Medical Biotechnologies and Translational Medicine, University of Milan, Milan, 20132, Italy

## Background

Somatic stem cells are the basic tools of regenerative medicine and gene therapy, providing unique opportunities for the therapy of genetic and acquired disorders. The molecular mechanisms underlying fundamental characteristics of human somatic stem cells, such as self-renewal, commitment and differentiation, are still poorly understood. A better knowledge of these mechanisms is crucial to the understanding of stem cell biology and to the development of stem cell-based therapies. The rapidly expanding information on the structural and functional characteristics of the human genome allows the development of genome-wide approaches to investigate the molecular circuitry wiring the genetic and epigenetic programs of somatic stem cells. High-throughput approaches are essential to study the transcriptome, the epigenome and the usage of regulatory elements in the genome.

## Materials and methods

The study presented here aims at defining the transcriptional and epigenetic profile of human hematopoietic stem/progenitor cells (HSPCs) and their committed, myeloid and erythroid progeny. For this purpose, high-throughput technologies were exploited such as:

- Cap Analysis of Gene Expression (CAGE), which allows genome-wide mapping of active promoters;

- Chromatin Immunoprecipitation coupled to deep sequencing technology (ChIP-Seq), for a genome-wide analysis of chromatin signatures typical of regulatory elements;

- Retroviral scanning, a novel technology based on the target site specificity of gamma-retroviral pre-integration complexes, to map transcriptionally active regulatory sequences.

## Results

CAGE analysis allowed to define >9,000 active promoters in HSPCs and their progeny. Around 500 transcripts were generated by the usage of alternative promoters, in some cases in a lineage-specific fashion. The different cell types shared most of the promoters, suggesting that the transcriptional state is largely maintained in early hematopoietic progenitors and precursors. Therefore, only a relatively small number of differentially used promoters defines the identity of hematopoietic cells at different stages of differentiation. 85% of the active promoters in each cell type were associated with known genes, whereas 13% of the transcripts identified by CAGE were classified as novel. Surprisingly, a significantly higher proportion (30%) of cell-specific promoters was not annotated. These novel transcripts are possibly involved in HSPC self-renewal, commitment and differentiation.

To obtain a genome-wide description of the transcriptional regulatory regions, we performed ChIP-seq analysis for histone methylations typical of active promoters and enhancers, H3K4me3 and H3K4me1, respectively. More than 15,000 putative promoters and 55,000 enhancers were discovered in all cell types analyzed. As expected, most of the promoters identified by CAGE overlapped with H3K4me3 peaks. Interestingly, a small fraction of TSSs was found in regions highly enriched in H3K4me1, indicating the presence of potential “enhancer RNA”.

Finally, retroviral scanning was used to map about 3000 activeenhancers involved in the control of growth, differentiation and development of hematopoietic cells. More than 60% of these regulatory regions were differentially used between HSPCs and committed precursors.

## Conclusions

Overall, this study provided an overview of the differential transcriptional programs of HSPCs and committed precursors and represents a unique source of genes and regulatory regions involved in self-renewal, commitment and differentiation of human hematopoietic stem cells and their progeny.

